# Moderate-Heavy Alcohol Consumption Lifestyle in Older Adults Is Associated with Altered Central Executive Network Community Structure during Cognitive Task

**DOI:** 10.1371/journal.pone.0160214

**Published:** 2016-08-05

**Authors:** Rhiannon E. Mayhugh, Malaak N. Moussa, Sean L. Simpson, Robert G. Lyday, Jonathan H. Burdette, Linda J. Porrino, Paul J. Laurienti

**Affiliations:** 1 Laboratory for Complex Brain Networks, Wake Forest School of Medicine, Winston-Salem, North Carolina, United States of America; 2 Neuroscience Program, Wake Forest School of Medicine, Winston-Salem, North Carolina, United States of America; 3 Department of Biostatistical Sciences, Wake Forest School of Medicine, Winston-Salem, North Carolina, United States of America; 4 Department of Radiology, Wake Forest School of Medicine, Winston-Salem, North Carolina, United States of America; 5 Department of Physiology and Pharmacology, Wake Forest School Medicine, Winston-Salem, North Carolina, United States of America; Indiana University, UNITED STATES

## Abstract

Older adults today consume more alcohol than previous generations, the majority being social drinkers. The effects of heavy alcohol use on brain functioning closely resemble age-related changes, but it is not known if moderate-heavy alcohol consumption intensifies brain aging. Whether a lifestyle of moderate-heavy alcohol use in older adults increased age-related brain changes was examined. Forty-one older adults (65–80 years) that consumed light (< 2 drinks/week and ≥ 1 drink/month, n = 20) or moderate-heavy (7–21 drinks/week, non-bingers, n = 21) amounts of alcohol were enrolled. Twenty-two young adults (24–35 years) were also enrolled (light, n = 11 and moderate-heavy, n = 11). Functional brain networks based on magnetic resonance imaging data were generated for resting state and during a working memory task. Whole-brain, Central Executive Network (CEN), and Default Mode Network (DMN) connectivity were assessed in light and moderate-heavy alcohol consuming older adults with comparisons to young adults. The older adults had significantly lower whole brain connectivity (global efficiency) and lower regional connectivity (community structure) in the CEN during task and in the DMN at rest. Moderate-heavy older drinkers did not exhibit whole brain connectivity differences compared to the low drinkers. However, decreased CEN connectivity was observed during the task. There were no differences in the DMN connectivity between drinking groups. Taken together, a lifestyle including moderate-heavy alcohol consumption may be associated with further decreases in brain network connectivity within task-related networks in older adults. Further research is required to determine if this decrease is compensatory or an early sign of decline.

## Introduction

As the baby boomer generation progresses into retirement age, research focused on cognitive decline is imperative as it carries one of the most costly personal, financial, and societal burdens associated with aging [1]. As cognitive decline has been shown to have many varying risk factors, with no single treatment or prevention proving effective, studying specific populations and risk factors is essential [2]. One potential risk factor requiring further study is social alcohol consumption. Social alcohol consumption is an increasingly common lifestyle choice in those 65 years or older and, as such, it is essential that we understand its impact on brain aging [3–5]. As these drinking patterns typically reflect a lifestyle choice, understanding their impact on brain functioning will help guide informed lifestyle decisions.

It has been well documented that brain functioning changes across the lifespan, with organization of functional connectivity decreasing with age [6]. Graph theory applied to neuroimaging has revealed that whole brain networks are made up of collections of smaller networks, or subnetworks, which are densely connected within themselves and sparsely connected to each other [7]. This shift in functional network organization in older adults suggests a breakdown in this pattern of high intra- and sparse inter-connectivity among the numerous networks within the brain. Some of these changes have been associated with maintenance or compensation of cognitive performance in aging adults [8] while others suggest it is related to cognitive decline [9, 10]. Although the cognitive implication of these brain changes continues to be debated, it is clear that desegregation of connectivity is associated with aging. It is vital that any studies evaluating the effect of alcohol on the aging brain examines connectivity within and between the subnetworks of the brain.

Little is known about how these age-related changes in brain organization may differ with varying levels of social alcohol consumption. Research focused on heavy alcohol consumption in younger adults suggest functional connectivity is sensitive to long-term heavy alcohol consumption [11]. Decreased default mode network (DMN) connectivity and increased central executive network (CEN) connectivity is associated with successful abstinence in alcoholics [12]. Disruption of DMN and CEN connectivity due to heavy alcohol use has been documented in younger [13, 14] and middle age adults [15, 16]. The functional connectivity of the DMN and CEN have also been shown to be sensitive to aging [17] with decreased connectivity within these networks being observed in older adults [8, 18, 19]. Given the vulnerability of these networks to the normal aging process, social alcohol consumption may accelerate age-related changes in functional brain connectivity.

Analysis of these functionally connected networks, such as the DMN and CEN, within the context of the whole brain network is becoming an increasingly utilized approach to understand brain changes [20]. An advantage of examining whole brain networks lies in the ability to capture connectivity of the entire brain. Thus, in addition to assessing connectivity between regions that belong to a network of interest, it is possible to evaluate how the network of interest connects to the remainder of the brain. To perform this type of analysis, the whole brain network is broken into network communities [21]. A network community consists of areas exhibiting high intra-connectivity with each other, but low inter-connectivity with the rest of the network. An evaluation of brain network communities readily reveals the DMN and CEN, particularly at rest and during working memory, respectively. The spatial location of the communities can be assessed to determine the extent to which a group’s network communities deviate from expected organization or how two groups differ in their network communities. With this approach, both integration (within-community connections) and segregation (external-community connections) of specific networks can be simultaneously evaluated and compared across age and alcohol consumption groups.

The overall goal of this study was to determine if a lifestyle of moderate-heavy alcohol consumption (7–21 drinks per week) in older adults increased age-related functional brain network changes during resting state and a working memory task. Primary comparisons of brain networks were made to a population of older adults that consumed low levels (less than 2 drinks per week and at least 1 drink per month) of alcohol. The main study findings were compared to brain networks from younger adults (24–35 years old) in order to contrast alcohol effects with age-related effects. Overall measures of whole brain connectivity were assessed using metrics such as *average degree* (extent of overall connectivity) and *efficiency* (ease of information flow). Community structure analysis of the DMN and CEN was also conducted.

Given the potential vulnerability of the aging brain to ethanol exposure, it was hypothesized that a lifestyle of moderate-heavy alcohol consumption in older age, compared to light alcohol consumption, would be associated with a decrease in community structure of the CEN during task and the DMN during rest.

## Materials and Methods

### Sample Demographics

Forty one older adults (65–80 years old) were recruited for the study. An additional 22 younger adults (24–35 years old) were recruited for purposes of age comparison. Participants were specifically recruited based on their alcohol consumption patterns, focusing on those reporting a lifestyle that included light or moderate-heavy alcohol consumption for at least the past 3 years. Light alcohol consumers were defined as those, on average, drinking less than 2 servings per week, but at least one serving per month. Moderate-heavy alcohol consumers were defined as those, on average, drinking 7–21 drinks per week without binging. Binging was defined as consumption exceeding 4 drinks for women or 5 drinks for men within 2 hours. It should be noted that those at the high end of the moderate-heavy group criteria would be considered above the threshold of risky drinking, especially in older adults (> 3 drinks per day or 7 drinks per week) [22]. To verify drinking patterns, daily alcohol intake over 3 months was measured using the Time Line Follow Back (TLFB) questionnaire [23]; a 12 oz. (beer), 5 oz. (wine), and a 1.5 oz. (hard liquor) glass were used to help represent standard drink sizes. Twenty older adults were light drinkers and 21 were moderate-heavy. Of the younger adults, 11 were light drinkers and 11 were moderate. These individuals were previously included in a recent report [24] evaluating results from a full cognitive assessment to measure the effect of moderate-heavy alcohol consumption on age-related cognitive decline. All participants were recruited via local advertisements or by word-of-mouth and were compensated for their participation. The Institutional Review Board of Wake Forest School of Medicine approved the study and all participants gave written informed consent.

### Screening

Participants were excluded if they did not report maintaining either a light or moderate-heavy alcohol consumption pattern on average for at least the past 3 years. They were also excluded if they reported more than 1 binge in the last 3 months (≥5 drinks for men and ≥4 drinks for women in less than 2 hours) [25]. Disqualification from the study included a > 0.00 alcohol reading (measured by an Intoxilyzer S—D5 breath alcohol screen, www.alcoholtest.com) or a positive test for cocaine, THC, opiates, amphetamines, methamphetamines or benzodiazepines (measured by an Alere iCassette 6 –panel drug screen, www.alere.com) at the start of any study visit. Subjects were excluded if they had a history of alcohol or drug abuse/dependence, current (within the last 6 months) Axis I disorders as determined by the Structured Interview for DSM Disorders (SCID) [26], or reported use of illicit drugs within the last 3 months. Typical daily consumption of caffeine and nicotine was also recorded for all enrolled participants as potential covariates.

In addition to alcohol and drug use exclusions, all subjects were evaluated for neurological and psychiatric conditions. Exclusion criteria included: a high risk for dementia measured by the Modified Mini Mental State Exam (3MSE score ≤ 80; Tables 1 & 2), active neurological dysfunction with the potential to affect cognitive processing (i.e. clinical diagnosis of: schizophrenia, Alzheimer’s disease, attention deficit-hyperactivity disorder, Parkinson’s disease, prior history of stroke, epilepsy, or mental retardation), or use of antipsychotic and/or antiepileptic medications. Those with serious CNS trauma (defined by a history of acquired sub—or epidural hematomas), previous brain surgery, or loss of consciousness for greater than 5 minutes were not included. Current use of antidepressants were allowed if treatment had stabilized for greater than 2 months and no active depression was exhibited according to the Center for Epidemiological Studies Depression Scale (CES-D) or Profile of Moods Survey (POMS) (see Tables 1 & 2). A body mass index (BMI, kg/m^2^) of < 18 (normal) or > 35 (moderately obese) was exclusionary to control for the interaction of weight and the metabolism of alcohol. Those with insulin dependent type I diabetes were also excluded, as were individuals with high blood pressure who had not had at least 1 year of stable treatment (see Tables 1 & 2). A positive pregnancy test, left handedness, corrected visual acuity < 20/40, and hearing loss was also exclusionary.

**Table 1:** Younger and Older Adult Group Demographics.

	YOUNGER ADULTS *(n = 22)*		OLDER ADULTS (*n = 41)*	
	*Mean ± SD*	*Min/Max*	*Mean ± SD*	*Min/Max*
**Age (years)**	*27*.*3 ± 3*.*4*	*24/35*	*70*.*6 ± 3*.*8*	*66/78*
**Sex (M|F)**	*10|12*	*n/a*	*22|19*	*n/a*
**Drinks / Day**	*0*.*9 ± 0*.*7*	*0*.*05/2*.*4*	*0*.*9 ± 0*.*9*	*0*.*04/2*.*7*
**Drinks / Week**	*6*.*2 ± 5*.*1*	*0*.*4/16*.*6*	*6*.*6 ± 6*.*2*	*0*.*10/18*.*7*
Maintenance of Alcohol Level (years)*	*5*.*0 ± 2*.*0*	*3/11*	*18*.*1 ± 14*.*6*	*2/51*
Total Alcohol Exposure (years)*	*8*.*3 ± 3*.*5*	*3/18*	*44*.*8 ± 10*.*7*	*10/60*
Dementia (3MSE)*	*98*.*7 ± 2*.*0*	*92/100*	*97*.*4 ± 3*.*2*	*87/100*
**Depression (CES-D)**	*4*.*8 ± 4*.*0*	*0/13*	*4*.*4 ± 4*.*3*	*0/19*
Education (years)*†	*19*.*1 ± 2*.*3*	*15/24*	*16*.*4 ± 2*.*5*	*12/25*
**Profile of Moods (POMS)**	*-0*.*6 ± 12*.*3*	*-14/27*	*-1*.*4 ± 14*.*1*	*-18/43*
**Antidepressant Medications (# of subjects)**	*none*		*see Table 2*	
Medical Conditions (# of subjects)**	*none*		*see Table 2*	
**Smoking**	*none*		*see Table 2*	
**Ethnicity/Race (# of subjects)**	*Asian (2) Black (1) Hispanic (2) White (16) Pacific Islander (1)*		*Asian (0) Black (2) Hispanic (0) White (39) Pacific Islander (0)*	

*p < 0.05 for null hypothesis of no difference between younger and older adults

**Listed medical conditions only include those with the potential to affect brain functioning.

^†^Majority of the younger adult group were current students

**Table 2:** Older Light and Moderate-Heavy Drinker Group Demographics.

	OLDER LIGHT DRINKERS *(n = 20)*		OLDER MODERATE-HEAVY DRINKERS *(n = 21)*	
	*Mean ± SD*	*Min/Max*	*Mean ± SD*	*Min/Max*
**Age (years)**	*71*.*1 ± 3*.*4*	*66/75*	*70*.*1 ± 4*.*2*	*66/78*
**Sex (M|F)**	*12|8*	*n/a*	*10|11*	*n/a*
Drinks / Day*	*0*.*2 ± 0*.*1*	*0*.*04/0*.*4*	*1*.*7 ± 0*.*6*	*0*.*7/2*.*7*
Drinks / Week*	*1*.*1 ± 0*.*7*	*0*.*1/2*.*8*	*11*.*8 ± 4*.*1*	*6*.*6/18*.*7*
**Maintenance of Alcohol Level (years)**	*21*.*0 ± 16*.*1*	*2/50*	*16*.*1 ± 14*.*4*	*4/51*
**Total Alcohol Exposure (years)**	*46*.*3 ± 10*.*0*	*15/60*	*43*.*4 ± 11*.*5*	*10/55*
**Dementia (3MSE)**	*97*.*1 ± 3*.*4*	*88/100*	*97*.*8 ± 3*.*0*	*87/100*
**Depression (CES-D)**	*4*.*5 ± 3*.*7*	*0/13*	*4*.*3 ± 5*.*0*	*0/19*
**Education (years)**	*16*.*2 ± 3*.*0*	*12/25*	*16*.*5 ± 2*.*1*	*12/20*
**Profile of Moods (POMS)**	*-3*.*4 ± 11*.*0*	*-18/22*	*0*.*5 ± 16*.*6*	*-16/43*
**Antidepressant Medications (# of subjects)**	Paroxetine (Paxil) (1)		Citalopram & Trazodone (1) Duloxetine (Cymbalta) (1) Escitalopram (Lexapro) (1) Mirtazapine (Remeron) (1)	
Medical Conditions (# of subjects)**	Cholesterol (4) Diabetes, non-insulin dependent (2) High blood pressure, stable with medication > 1 year (7)		Cholesterol (3) Diabetes, non-insulin dependent (1) High blood pressure, stable with medication > 1 year (10)	
**Smoking (# of subjects)**	Cigarettes, < 30/day (1) Ex-smokers (0)		Cigarettes, < 30/day (0) Ex-smokers (1)	
**Ethnicity/Race (# of subjects)**	*Asian (0) Black (1) Hispanic (0) White (19) Pacific Islander (0)*		*Asian (0) Black (1) Hispanic (0) White (20) Pacific Islander (0)*	

*p < 0.05 for null hypothesis of no difference between light and moderate-heavy older adult drinkers

**Listed medical conditions only include those with the potential to affect brain functioning.

### Image Collection

Participants arrived approximately 1 hour prior to MR scanning and were briefed on safety and imaging protocols. The imaging protocol was performed in the following order: a T1-weighted structural scan, a blood-oxygen-level-dependent (BOLD)-weighted resting scan, and a BOLD-weighted 1-back task performance scan. A 2-back scan was also collected, but excluded from the final analysis as half of the older adults (both drinking groups) scored around 50% accuracy. As there is a 50% chance of guessing correctly during this task, confidence in the engagement of working memory versus simply guessing was low. This decision was supported by Kirchner et al. [27], that reported a high percentage of older adults misunderstanding the task or simply giving up. Participants were told to fixate on a cross that was projected onto a screen during resting scans. Percent accuracy achieved on the 1-back task was used as a measure of working memory [27]. Participants were presented with white letters one at a time on a black background. After the first letter was presented, individuals responded with either a right (yes) or left (no) finger press to indicate whether or not the letter they were viewing was identical to the one that preceded it. The task lasted 6 minutes and consisted of 120 trials. Each trial lasted 3,000 milliseconds. A letter was presented for the first 300 milliseconds and was followed by a blank black screen for 2,700 milliseconds. Participants could respond at any point during a trial. Participants were given time to practice the task for 1 minute outside and 1 minute inside of the scanner.

MRI data were obtained on a 3T Siemens Skyra equipped with a 32-channel head coil, a rear projection screen, and both left and right hand response boxes. High-resolution (0.98 x 0.98 x 1.0 mm) T1-weighted structural scans were acquired in the sagittal plane using a single-shot 3D MPRAGE GRAPPA2 sequence (acquisition time = 5 minutes and 30 seconds, TR = 2.3 seconds, TE = 2.99 ms, 192 slices). BOLD images were taken during resting state (eyes open with fixation point) and 1back task. Both BOLD-weighted image sequences were acquired in the transverse plane using an echo-planar imaging sequence (resolution = 3.75 x 3.75 x 5.0 mm acquisition time = 6 minutes and 20 seconds, TR = 2.0 seconds, TE = 25 ms, flip angle = 75°, 35 slices per volume, 187 volumes). The first 20 seconds (10 image volumes) were discarded to allow signal to achieve equilibrium.

### Image Preprocessing

Image pre-processing was performed using SPM8 software (www.fil.ion.ucl.ac.uk/spm/). Structural image data were first skull-stripped, then segmented into maps (gray matter, white matter, and cerebrospinal fluid) and normalized to MNI template space (Montréal Neurological Institute, www.mni.mcgill.ca) using the unified segmentation algorithm [28]. BOLD-weighted (functional) image data were realigned to the first volume and slice-time corrected. Functional image data were then co-registered to the skull-stripped structural image in native space. The normalization parameters derived from the unified segmentation of the structural image were then applied to the functional images. Physiological noise and low frequency drift were reduced by regressing out 3 mean signals (whole-brain, white matter, and cerebrospinal fluid) and band-pass filtering (0.009–0.08 Hz) the images. This global signal adjustment was done to help correct for the positive correlational bias exhibited by many brain areas to this global signal, allowing for greater regional signal detection [29]. The mean signal from a small region of interest in the superior sagittal sinus was also included in the regression analysis. This was done to avoid the introduction of false correlations caused by pulsating blood drainage in this area. Functional image data were also motion corrected using a protocol designed to eliminate scan volumes with both excessive frame-wise displacement and BOLD signal change [30]. The number of participants that required motion correction did not differ significantly between groups. However, for those that did require motion correction, a greater average number of volumes were removed from the older adult group with the majority (67%) occurring during the 1back task. This was expected as the 1back task requires hand movement during the keypad answer selection.

### Generating Whole-Brain Functional Networks

Voxel-based, whole-brain networks were generated from pre-processed functional image data for each participant. Network data was restricted to gray matter voxels as defined by the Automated Anatomical Labeling (AAL) atlas [31]. After regressing out confounding signals, a Pearson correlation was performed between time series for each voxel pair. Thus, each image voxel served as a network node and the network connections were represented by similarities in the voxels’ time-courses (for details see [32]). Correlation matrices were then thresholded to maintain consistent edge density across subjects. This process ensured that comparisons between networks were of equivalent density relative to the number of network nodes. This was done using the formula N = K^s^ [33], with N equal to the number of nodes and K equal to average degree (number of edges per node in the network). Networks were thresholded at S = 2.5 based on work showing that brain networks fragment when S > 3 [34] and that the reproducibility is highest with an S between 2 and 3 [35]. Correlation values equal to or greater than the correlation coefficient solving the threshold formula were set to 1 and all others set to 0 to produce undirected, unweighted networks. The resulting whole-brain networks from each study participant then served as the basis for all further analyses.

### Whole-Brain Summaries of Functional Network Topology

The individual functional networks were analyzed for differences in whole brain connectivity. A brief description of network metrics utilized in this study follows (see [36] for a detailed review). Degree (K) was used as a measure of connectivity and is equal to the number of links connected to a node. Efficiency is a measure of how easily information can transfer within a network [37]. Values for local efficiency (E_loc_) range from 0 to 1, where a value of 1 represents a node whose neighbors are entirely interconnected (i.e. maximum regional specificity). Values for global efficiency (E_glob_) also range from 0 to 1, where a value of 1 represents a node directly linked to all other nodes in the network (i.e. maximum distributive processing). The metrics K, E_loc_, and E_glob_ were first calculated at the individual-node level. Values from all nodes within the network were averaged in order to generate whole brain summaries.

### Functional Brain Network Community Structure

Community structure analyses were performed on the networks from each study participant. A community is a group of nodes within a network that exhibit high intra-connectivity with each other, but low inter-connectivity with other nodes in the network. The individual whole brain functional networks were first partitioned into communities utilizing modularity (Q) as a quantitative measure [21, 38] and the Louvain method optimized the modularity of a network’s partitions [39]. This process was repeated 10 times for each individual’s network and the partition representing the best solution (i.e. highest Q value) was chosen for further analysis.

Once the communities for each study participant were identified, we assessed the spatial overlap between the communities to determine the population consistency. Scaled inclusivity (SI) was used to quantitatively assess the spatial overlap of communities across individuals [40]. This statistic considers overlap and disjunction between all modules in a whole-brain analysis and does not require subjective determination of module pairs across individuals. Brain images resulting from this analysis depict the consistency of a community across subjects including similarity of both location and size [41, 42].

**Fig 1:**
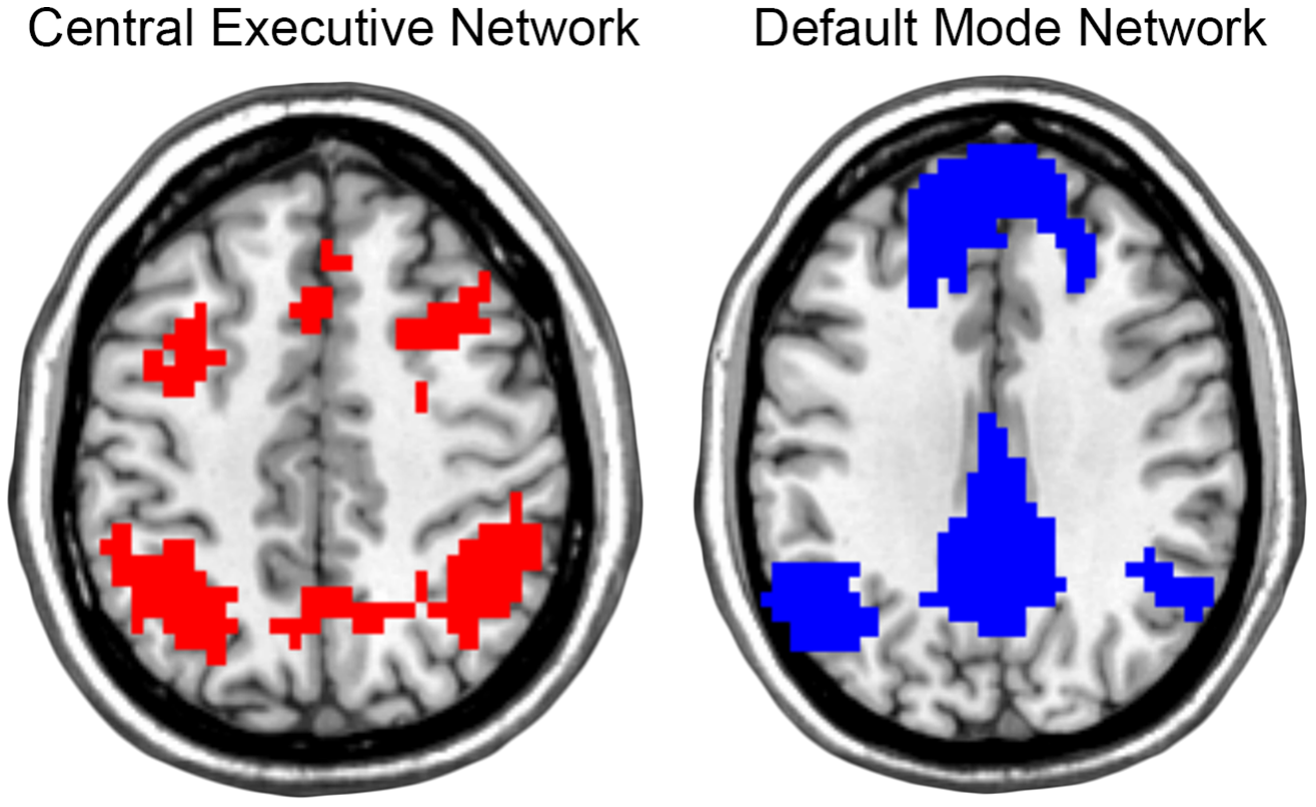
Templates for the Central Executive Network and Default Mode Network. The central executive network template (red) included bilateral portions of the dorsal lateral prefrontal cortex, superior medial frontal cortex, and the superior parietal cortex. The default mode network template (blue) included bilateral portions of the medial prefrontal cortex, posterior cingulate cortex, precuneus, and inferior parietal cortex (MNI z-coordinates: CEN = 46mm, DMN = 34mm). These templates were used in the SI analysis of community structure.

In the current project, SI was used to determine the extent to which participant’s communities aligned with brain regions associated with the central executive network (CEN) and the default mode network (DMN). Each of these brain networks has been characterized in a host of studies (see [43] theoretical review of these networks). The specific CEN and DMN templates used in this study (Fig 1) were based on network partitions detected during repeated resting-state and 1back task conditions from an independent study [44]. These templates reflect the *actual* functional communities present during the 1 back working memory task (CEN template) and during rest (DMN template). Any overlap between the template images was removed from both templates to ensure specificity of the analysis. For each participant, the brain network community structure was quantitatively assessed with SI to determine the spatial consistency with the CEN and DMN templates. The SI values from all voxels encompassed by the CEN and DMN templates were then averaged for each subject and used for statistical analyses.

### Statistics

The relationships between alcohol consumption (light, moderate-heavy) and condition (rest, task) with whole-brain summaries of network topology and community consistency were assessed using double multivariate analyses of covariance (DMANCOVAs) tests. Confounding variables included total number of years consuming alcohol, years the individual maintained either a light or moderate-heavy alcohol consumption pattern, and hypertension due to the BOLD signal’s dependence on neurovascularization [45]. BMI was also treated as a confound due to its association with blood alcohol concentration [46], as well as it’s relation to decreased brain volume [47, 48]. If sex-differences were non-significant; age, alcohol consumption, and condition were collapsed across sex. Non-normally distributed variables were appropriately transformed and tests of normality were performed for each set of dependent variables (given the covariates). Model validity was ensured by co-linearity and outlier assessments. If a significant main effect of age, alcohol consumption or condition was found; step-down tests of the least square means were performed employing Tukey’s adjustment for multiple comparisons. Estimated means and 95% confidence intervals are presented, and SAS software version 9.3 was utilized for all statistical analyses.

## Results

### Demographics

#### Younger and Older Adults

Younger adults (n = 22) were on average 27.3 ± 3.4 years old and older adults (n = 41) were 70.61 ± 3.8 years old. Younger and older adults were balanced in terms of the number of males and females within each group and scored similarly on measures of depression, mood, and dementia. Age groups consumed a similar number of alcoholic drinks per day (younger: 0.89 ± 0.73; older: 0.93 ± 0.88) and per week (younger: 6.2 ± 5.1; older: 6.6 ± 6.2 drinks). Older adults consumed alcohol for a greater number of years than younger adults. This was expected given the difference in age and analyses were corrected for this fact. See Table 1 for details.

#### Older Adults—Light and Moderate-Heavy Drinkers

Light drinkers (n = 20) were on average 71.1 ± 3.4 years old and moderate-heavy drinkers (n = 21) were 70.1 ± 4.2 years old (Table 2). These groups were balanced in terms of the number of males and females in each group. Moderate-heavy drinkers consumed significantly more alcoholic drinks per day (1.7 ± 0.6) than light drinkers (0.2 ± 0.1). Moderate-heavy drinkers also consumed significantly more alcoholic drinks per week (11.8 ± 4.1 drinks) than light drinkers (1.1 ± 0.7 drinks). Careful enrollment ensured that important factors related to alcohol drinking history were not significantly different, including the number of years each group had maintained their alcohol consumption level, and the total number of years they had been drinking alcohol. Both groups scored similarly on measures of depression (CES-D), mood (POMS), and dementia (3MSE). Physiological factors including high blood pressure and BMI were also balanced. Sixteen out of twenty light drinkers were on a stable regimen of prescription medication. This included seven individuals on medication to treat high blood pressure, four individuals on medication to treat high cholesterol, two individuals on medication to treat Type II diabetes, and one individual on anti-depressants. Eighteen out of twenty-one moderate-heavy drinkers were on a stable regimen of prescription medication. This included ten individuals on medication to treat high blood pressure, three individuals on medication to treat high cholesterol, one individual on medication to treat Type II diabetes, and four individuals on anti-depressants. Other commonly cited conditions that required prescription medication included hypothyroidism, arthritis, osteoporosis, and heartburn. One light drinker smoked (less than thirty cigarettes per day), and there were no ex-smokers. There were no moderate-heavy drinkers that actively smoked cigarettes; one individual was an ex-smoker.

### Working Memory Task Performance

#### Younger vs. Older Adults

Working memory (1-back) percent accuracy for younger adults resulted in an estimated mean 98.31%, 85.95–100%. Older adults’ estimated mean was 89.61%, 82.30–96.92%. Younger adults significantly outperformed older adults when the statistical model was not corrected for confounding variables (t (1, 61) = 2.36, p = 0.02). Significance was lost when confounding variables (presence or absence of high blood pressure, BMI, total number of years consuming alcohol, and total number of years of alcohol pattern maintenance) were included in the final statistical model (F (5, 57) = 1.22, p = 0.24). These behavioral results were complemented by a full battery of cognitive tests collected on a previous study visit and both were reported by [24].

#### Older Adults—Light vs. Moderate-Heavy Drinkers

Working memory (1-back) percent accuracy was not significantly different between light and moderate-heavy drinkers after correcting for confounding variables (presence or absence of high blood pressure, BMI, total number of years consuming alcohol, and total number of years of alcohol pattern maintenance) (F (5, 35) = 1.35, p = 0.25). Light drinkers’ percent accuracy resulted in an estimated mean of 92.85%, 83.84%– 100%; moderate-heavy drinkers scored 86.38%, 78.05–94.70%. These behavioral results were complemented by a full battery of cognitive tests collected on a prior study visit and have been previously reported in detail [24].

### Whole-Brain Summaries of Functional Network Topology

#### Younger vs. Older Adults

A significant effect of age (F (4, 54) = 3.99, p = 0.007) and condition (F (4, 54) = 5.08, p = 0.002) on whole-brain summaries of network topology was shown. No interaction of drinking pattern and condition was observed (F (4, 54) = 1.24, p = 0.305). Although statistical significance was not reached, networks were more connected in older adults than in younger adults (K; t (57) = -1.80, p = 0.08) during rest. Older adults at rest had significantly greater global efficiency than younger adults (Eglob; t (57) = -2.35, p = 0.02) but similar levels of local efficiency (Eloc; t (57) = 0.81, p = 0.42). In the task condition, networks were more connected in older adults than in younger adults (t (57) = -1.77, p = 0.08), but this difference did not reach significance. Networks in the task condition showed similar levels of global efficiency in both younger and older adults (t (57) = 0.04, p = 0.97) and local efficiency was greater (approached significance) in younger adults than in older adults (t (57) = 1.75, p = 0.08). Estimated means and 95% confidence intervals for whole-brain summaries of network topology in younger and older adults are presented in Table 3.

**Table 3:** Younger and Older Adult Group Whole-Brain Summaries of Network Structure.

	YOUNGER ADULTS *(n = 22)*		OLDER ADULTS *(n = 41)*	
	*Estimated Mean*	*95% CI*	*Estimated Mean*	*95% CI*
**Degree (K)—Rest**	*52*.*51*	*51*.*84–53*.*19*	*53*.*41*	*53*.*01–53*.*81*
**Degree (K)—Task**	*52*.*52*	*51*.*84–53*.*20*	*53*.*40*	*53*.*00–53*.*80*
**Local Efficiency (E**_**loc**_**)—Rest**	*0*.*51*	*0*.*49–0*.*54*	*0*.*50*	*0*.*49–0*.*51*
**Local Efficiency (E**_**loc**_**)—Task**	*0*.*52*	*0*.*50–0*.*54*	*0*.*49*	*0*.*48–0*.*50*
**Global Efficiency (E**_**glob**_**)—Rest**	*0*.*16*	*0*.*14–0*.*19*	*0*.*20*	*0*.*19–0*.*22*
**Global Efficiency (Eglob)—Task**	*0*.*22*	*0*.*19–0*.*24*	*0*.*22*	*0*.*20–0*.*23*

#### Older Adults—Light vs. Moderate-Heavy Drinkers

The whole-brain functional network measures did not differ between drinking groups (Table 4). DMANCOVA analysis of network topology metrics (local and global efficiency, degree) revealed no significant effect of drinking pattern (F (4, 32) = 0.40, p = 0.80) nor condition (F (4, 32) = 1.59, p = 0.20). No interaction of drinking pattern and condition was observed (F (4, 32) = 0.63, p = 0.65). Estimated means and 95% confidence intervals of the whole-brain network for both drinking groups are reported in Table 4. Together, these results suggest that social alcohol consumption levels do not impact overall brain connectivity at a local or global scale during rest or working memory task.

**Table 4:** Older Light and Moderate-Heavy Drinker Group Whole-Brain Summaries of Network Structure.

	OLDER LIGHT DRINKERS *(n = 20)*		OLDER MODERATE-HEAVY DRINKERS *(n = 21)*	
	*Estimated Mean*	*95% CI*	*Estimated Mean*	*95% CI*
**Degree (K)—Rest**	*53*.*18*	*52*.*78–53*.*58*	*53*.*12*	*52*.*73–53*.*51*
**Degree (K)—Task**	*53*.*18*	*52*.*78–53*.*58*	*53*.*12*	*52*.*72–53*.*51*
**Local Efficiency (E**_**loc**_**)—Rest**	*0*.*50*	*0*.*49–0*.*51*	*0*.*50*	*0*.*49–0*.*51*
**Local Efficiency (E**_**loc**_**)—Task**	*0*.*49*	*0*.*48–0*.*50*	*0*.*50*	*0*.*48–0*.*51*
**Global Efficiency (E**_**glob**_**)—Rest**	*0*.*20*	*0*.*18–0*.*21*	*0*.*20*	*0*.*19–0*.*22*
**Global Efficiency (Eglob)—Task**	*0*.*22*	*0*.*21–0*.*24*	*0*.*22*	*0*.*21–0*.*24*

### Functional Brain Network Community Structure

#### Younger vs. Older Adults

**Fig 2:**
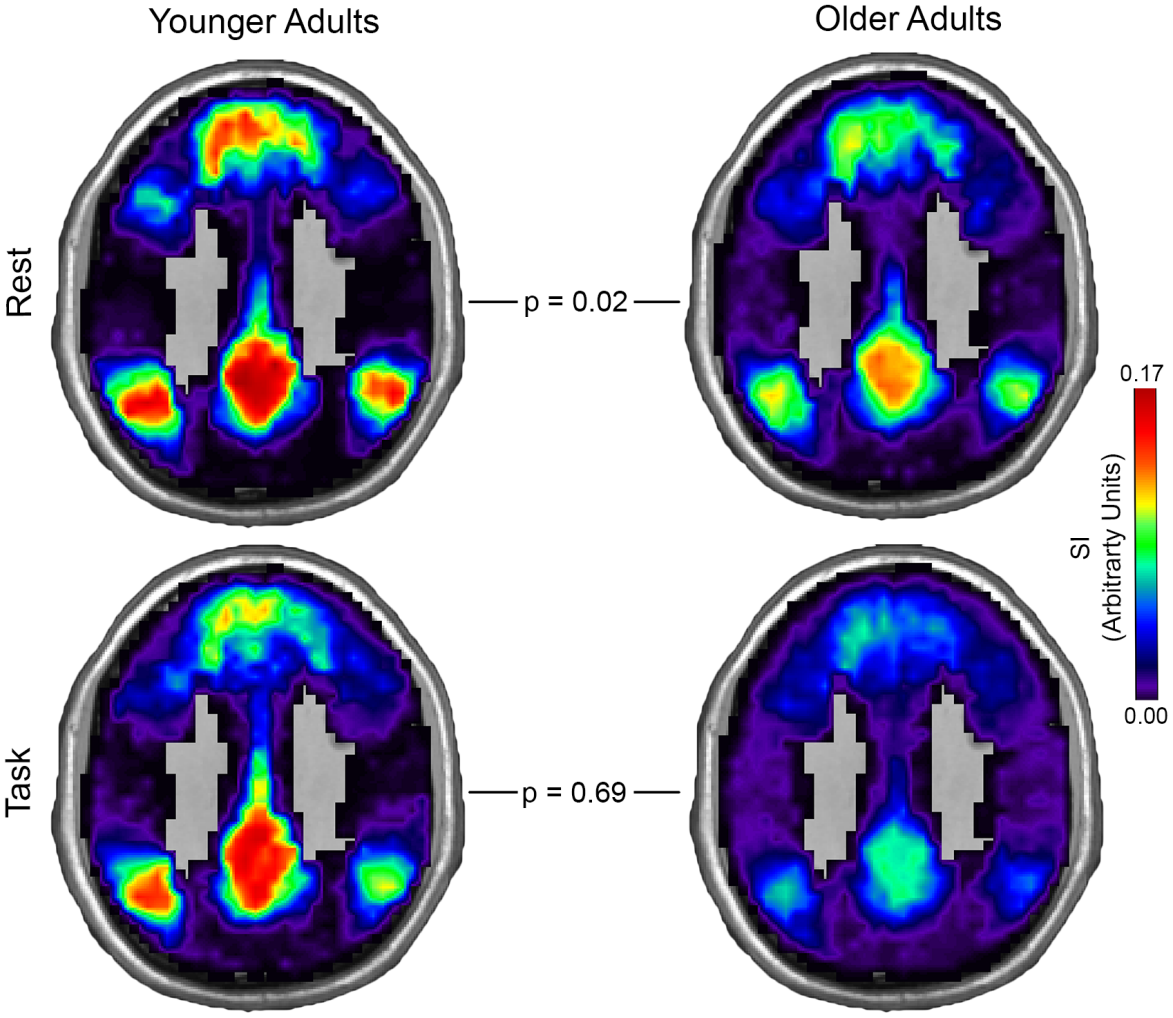
Default Mode Network Community Consistency in Younger and Older Drinkers. Spatial consistency of these communities was assessed using the scaled inclusivity (SI) metric. Maximum spatial overlap of these communities (higher SI values) was represented as warmer colors and minimum overlap represented by cooler colors. Default Mode Network community consistency was significantly higher in younger adults at rest, but did not differ by age group during task (MNI z-coordinate = 33mm).

**Fig 3:**
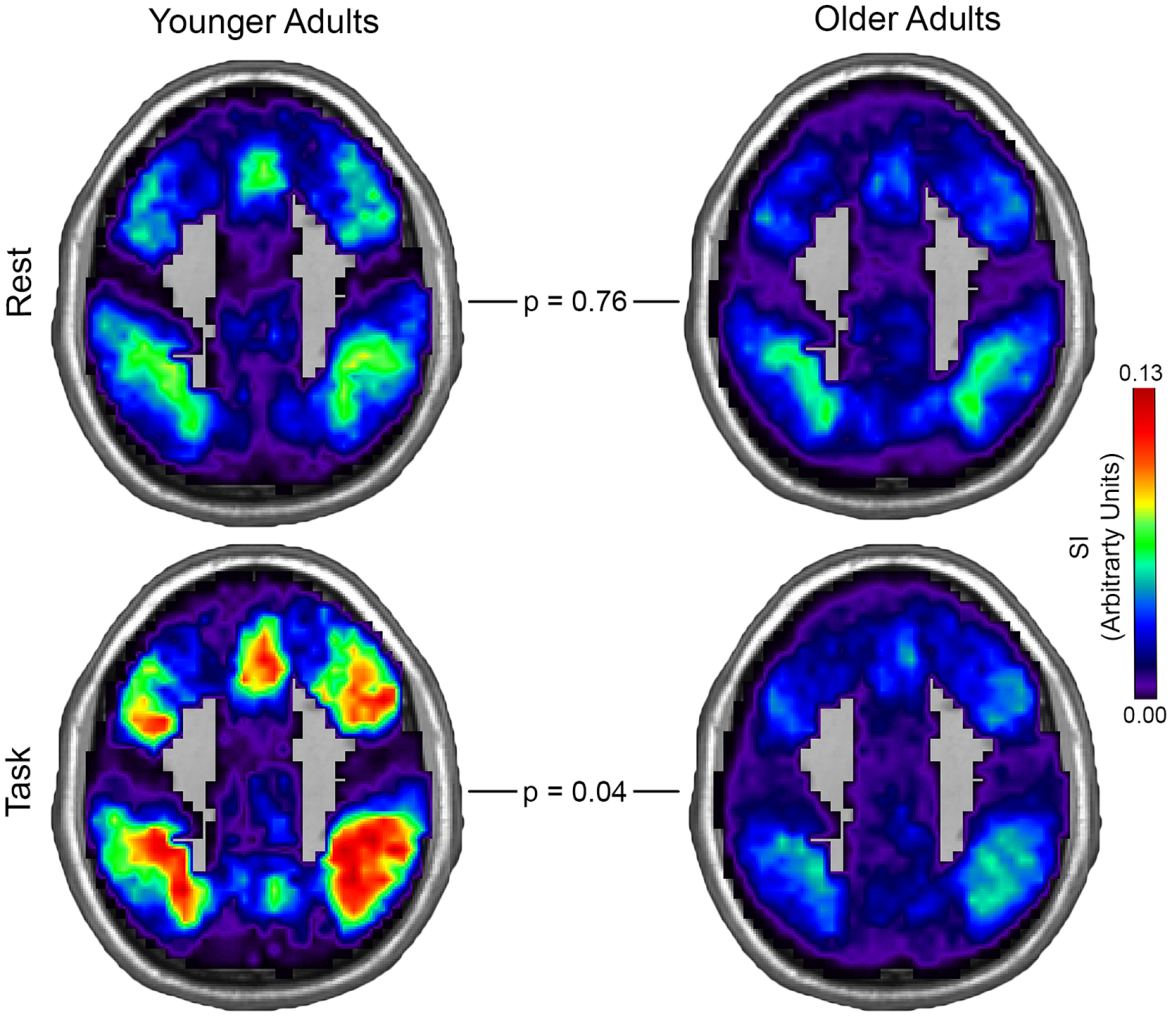
Central Executive Network Community Consistency in Younger and Older Drinkers. Central Executive Network community consistency was similarly low in both younger and older adult groups during rest, but was significantly lower in older adults in the task condition. Spatial consistency of these communities was assessed using the scaled inclusivity (SI) metric (see explanation in Fig 2). Note that the max SI value differs from previous figures and the color bar is scaled appropriately (MNI z-coordinate = 42mm).

Younger and older adult community consistency maps for the default mode network (DMN) and central executive network (CEN) in both rest and task conditions are shown in Figs 2 and 3. There was no overall effect of age (F (2, 56) = 1.73, p = 0.19). However, condition (F (2, 56) = 2.95, p = 0.06) and the interaction of age and condition (F (2, 56) = 2.87, p = 0.07) showed a near significant effect. Older and younger adults did not differ in CEN community consistency during rest (t (57) = 0.30, p = 0.76). However, the CEN community consistency was significantly lower in older adults during the task (t (57) = 2.11, p = 0.04). DMN community consistency was lower in older adults during rest (t (57) = 2.42, p = 0.02), but no difference between age groups was observed during task (t (57) = 0.40, p = 0.69). Estimated means and 95% confidence intervals for DMN and CEN community consistency in younger and older adults is presented in Table 5.

**Table 5:** Younger and Older Adult Group Consistency of Default Mode Network and Central Executive Network Communities at Rest and in Task.

	YOUNGER ADULTS *(n = 22)*		OLDER ADULTS *(n = 41)*	
	*Estimated Mean*	*95% CI*	*Estimated Mean*	*95% CI*
**DMN SI—Rest**	*0*.*10*	*0*.*07–0*.*13*	*0*.*05*	*0*.*03–0*.*07*
**CEN SI—Rest**	*0*.*05*	*0*.*03–0*.*06*	*0*.*04*	*0*.*03–0*.*05*
**DMN SI—Task**	*0*.*08*	*0*.*05–0*.*12*	*0*.*07*	*0*.*05–0*.*10*
**CEN SI—Task**	*0*.*08*	*0*.*05–0*.*10*	*0*.*03*	*0*.*02–0*.*05*

#### Older Adults—Light vs. Moderate-Heavy Drinkers

**Fig 4:**
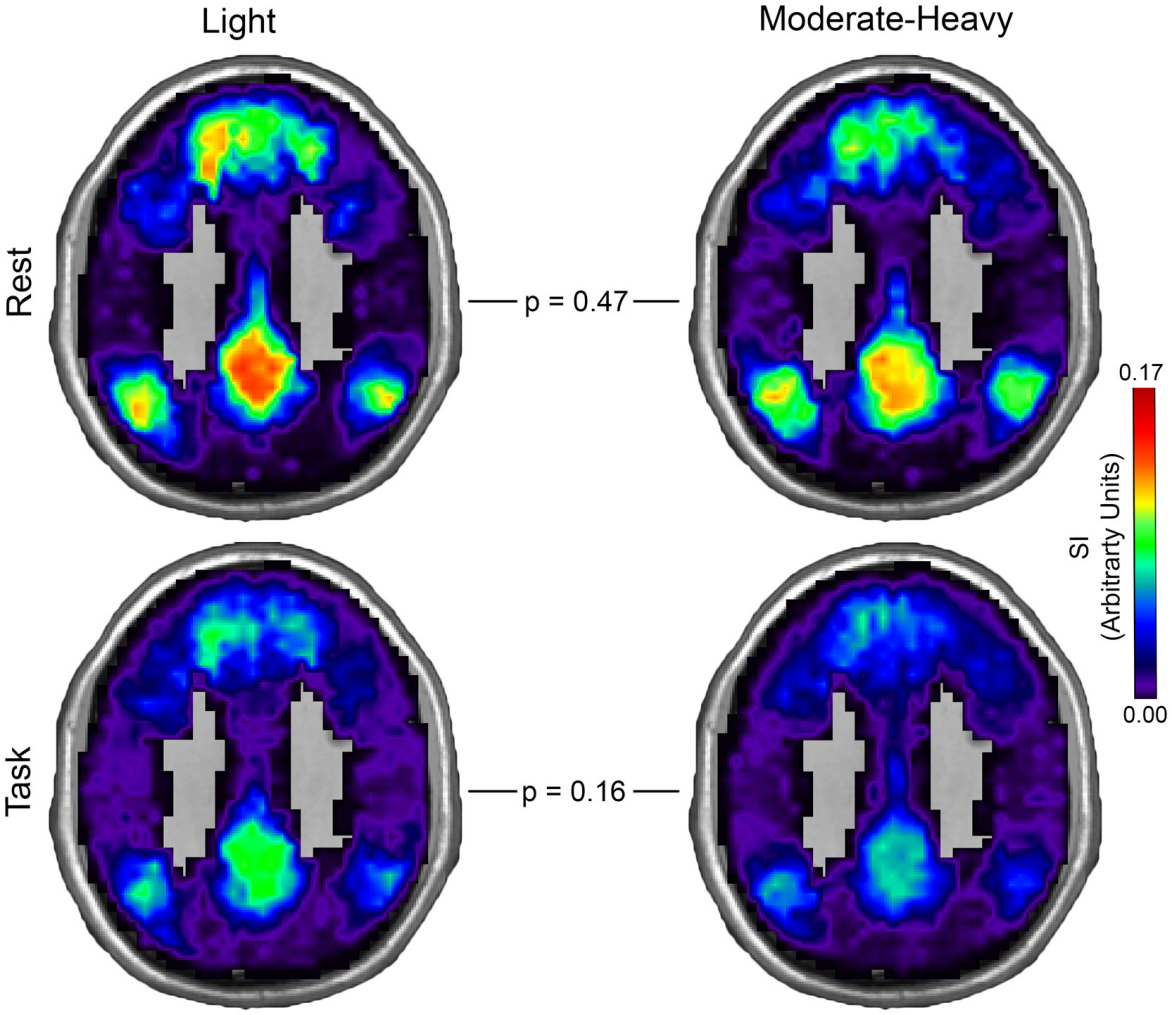
Default Mode Network Community Consistency in Light and Moderate-Heavy Drinkers. Spatial consistency of these communities was assessed using the scaled inclusivity (SI) metric. Maximum spatial overlap of these communities (higher SI values) was represented as warmer colors and minimum overlap represented by cooler colors. Default Mode Network community consistency was similar between light and moderate-heavy drinkers. Results pointed to decreased consistency in moderate-heavy drinkers compared to light drinkers, but the differences did not achieve significance (MNI z-coordinate = 33mm).

**Fig 5:**
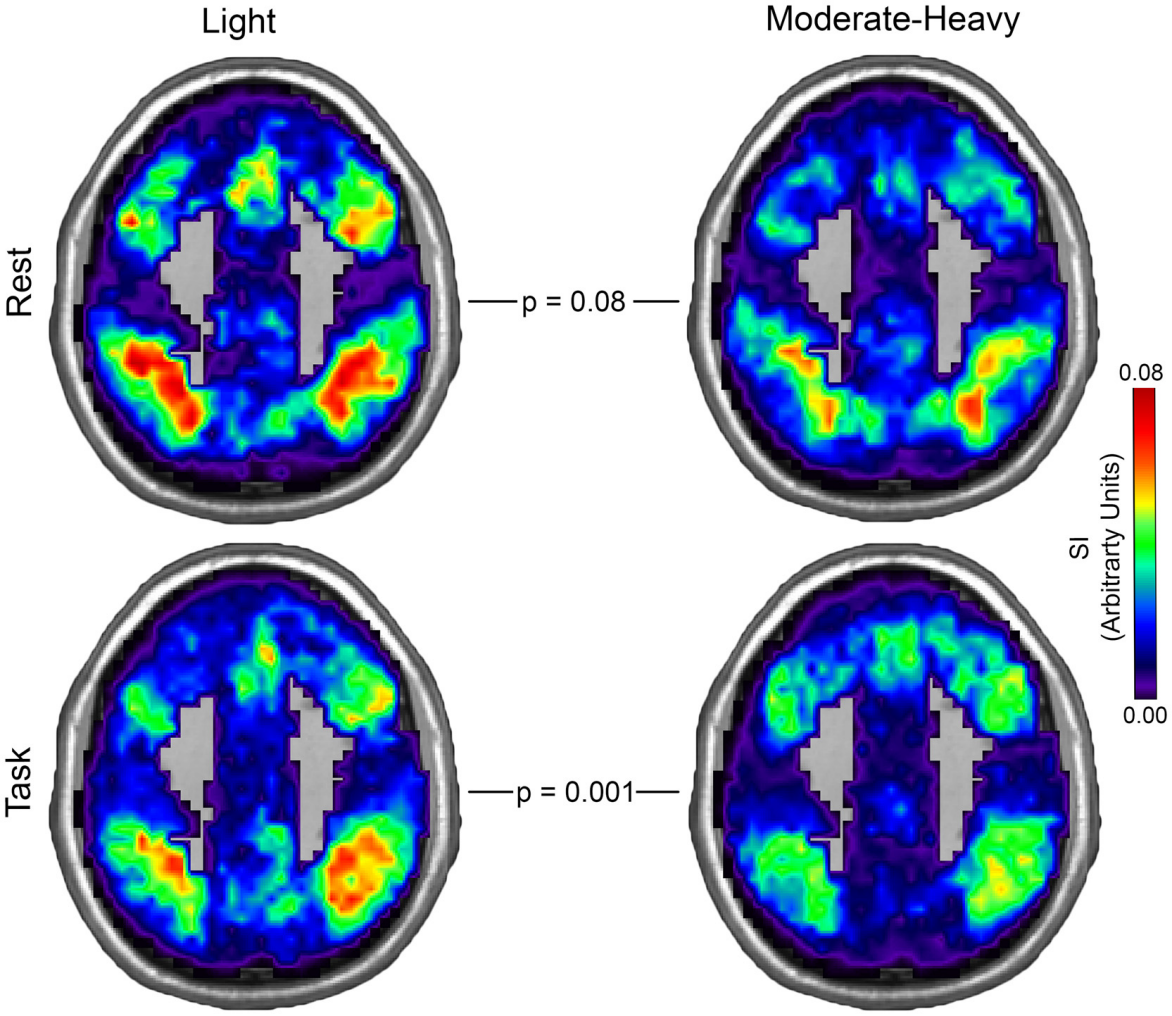
Central Executive Network Community Consistency in Light and Moderate-Heavy Drinkers. Central Executive Network community consistency was lower in moderate-heavy than in light drinkers during rest and statistically lower when in engaged in the task. Spatial consistency of these communities was assessed using the scaled inclusivity (SI) metric (see explanation in Fig 4). Note that the max SI value is lower relative to Fig 4 and the color bar is scaled appropriately (MNI z-coordinate = 42mm).

Light and moderate-heavy drinker community consistency maps for the DMN and CEN in both rest and task conditions are shown in Figs 4 and 5. Alcohol consumption level significantly affected overall community consistency (F (2, 32) = 7.90, p = 0.002). There was no effect of condition (F (2, 32) = 0.44, p = 0.65) nor was an interaction of alcohol consumption level and condition observed (F (2, 32) = 1.11, p = 0.34). In the rest condition, DMN community consistency was similar between groups (t (35) = 0.89, p = 0.38) while higher CEN community consistency in light drinkers (t (35) = 1.79, p = 0.08) approached significance. In the task condition, CEN community consistency was significantly higher in light drinkers than in moderate-heavy drinkers (t (35) = 3.40, p = 0.002) while DMN community consistency was similar between groups (t (35) = 1.43, p = 0.16). Estimated means and 95% confidence intervals for DMN and CEN community consistency is presented in Table 6.

**Table 6:** Older Light and Moderate-Heavy Drinker Group Consistency of Default Mode Network and Central Executive Network Communities at Rest and in Task.

	OLDER LIGHT DRINKERS *(n = 20)*		OLDER MODERATE-HEAVY DRINKERS *(n = 21)*	
	*Estimated Mean*	*95% CI*	*Estimated Mean*	*95% CI*
**DMN SI—Rest**	*0*.*08*	*0*.*06–0*.*10*	*0*.*07*	*0*.*05–0*.*09*
**CEN SI—Rest**	*0*.*05*	*0*.*03–0*.*06*	*0*.*03*	*0*.*02–0*.*04*
**DMN SI—Task**	*0*.*06*	*0*.*04–0*.*07*	*0*.*04*	*0*.*02–0*.*05*
**CEN SI—Task**	*0*.*04*	*0*.*03–0*.*05*	*0*.*02*	*0*.*01–0*.*03*

## Discussion

This study focused on social alcohol consumption in older adults and tested whether or not a lifestyle of moderate-heavy alcohol consumption was associated with increased age-related brain network changes compared to young adults. An analysis of overall network topology as measured by degree, global efficiency, and local efficiency revealed that alcohol consumption levels were not associated with overall changes in functional brain network topology in older adults. However, topology did differ when older adults were compared to younger adults. During rest, older adults exhibited significantly greater global efficiency than younger adults with overall connectivity (degree) approaching statistical significance. During task, greater overall connectivity and lower local efficiency compared to that in younger adults was observed, although these results only approached significance. Taken together, the findings suggested decreased segregation of connectivity in older adults compared to younger adults. These results are in line with previous work describing decreased segregation of network connectivity in aging populations [10].

A more focused analysis of connectivity of the default mode network (DMN) and central executive network (CEN) using community structure helped elucidate effects that were not captured by whole brain network topology. This analysis measured the consistency with which brain areas were members of specific communities across the groups. This method is ideal for assessing decreases in large-scale segregation of brain functioning without losing the impact of individual variability. It is important to note that this approach is not limited to connectivity of one particular area, such is the case with more traditional “seed” based connectivity analysis [49]. When using whole-brain networks, *every* region (voxels in our case) is treated as its own “seed” allowing for connectivity of the entire brain to be captured, not just that from a specific region. The community structure then serves to focus the analysis to investigate connectivity within specific networks, such as the DMN and CEN. Therefore, these results not only provide insight to functional changes occurring within the DMN and CEN, but also capture altered connectivity to brain areas external to these networks. This insight is essential if we are to understand patterns of large scale brain plasticity thought to occur with alcohol use and aging.

An effect of age and moderate-heavy drinking was observed when analyses of specific network communities (DMN and CEN) were performed. Compared to younger adults, older adults exhibited decreased DMN community structure consistency during rest. This observation was not surprising as previous work revealed decreased connectivity in this network in aging populations [8, 17]. Alcohol consumption did not affect DMN consistency in older adults. The CEN community in older adults did not differ from young adults during rest. However, a significant decrease in community structure was observed in older adults when participants were engaged in the working memory task. Further differences in CEN community consistency were observed between light and moderate-heavy older adult drinkers. A near significant decrease in community structure was observed in the moderate-heavy drinkers even at rest. This decrease became significant during the working memory task. Thus, the findings indicate that the older moderate-heavy drinkers exhibit an enhanced age-related decline in CEN community structure.

Extensive research on age-related brain changes has led to two overarching hypotheses: compensation and dedifferentiation [50]. Compensation reflects resilience to aging by expanding functional connectivity beyond that which is typically observed in young, healthy populations. In contrast, dedifferentiation views this loss of specific functional organization observed in youth as a sign of unhealthy aging and decline. Of course, the implications of brain changes manifest behaviorally. Behavioral performance declines that are associated with brain changes lend support to the dedifferentiation hypothesis. Changes in DMN and CEN brain regions have been specifically associated with memory and attention decline in aging, with some even occurring before symptoms have become apparent [51]. For example, it has been demonstrated that both DMN and CEN functional correlations were greatly reduced in aging during a semantic-decision task and that these reductions were associated with cognitive decline [18]. Another study investigating functional connectivity in the aging brain showed expanded distribution of brain activity during a working memory task, with changes specifically focused on frontal-parietal connectivity [52]. This recruitment of additional resources has been demonstrated specifically during the 1back working memory task in an elderly population [53].

Although this study alone cannot resolve the compensation vs. dedifferentiation dilemma, we explore both hypotheses to place the current study findings within this context. The compensation hypothesis may be supported as this population of healthy older adults did not perform worse than the younger adults on the working memory task, but they did exhibit reorganization of functional connectivity in the CEN during task performance. The CEN results demonstrate that a lifestyle of moderate-heavy alcohol consumption did, in fact, increase age-related declines in community structure. The declines were most clearly revealed when engaged in the working memory task. The strong effect observed during task is especially of interest, as this community is known to engage during goal-directed cognitive tasks in healthy populations [43, 54]. This decrease in community structure suggests that brain areas simultaneously engaged during effective cognitive processing in healthy populations are less connected with each other and more connected with other brain areas in these moderate-heavy alcohol consumers. Previous work has described compensatory brain functioning in aging as a result of deficits occurring in areas typically sourced in healthy young adults during tasks [8]. These findings may suggest that moderate-heavy alcohol consumption increases the need for compensatory brain connectivity beyond that observed in healthy aging. Connectivity of the DMN in the older adults was similar in the two alcohol consumption groups, suggesting that a moderate-heavy drinking lifestyle did not have a negative impact on the aging process in this circuit.

However, the current study design cannot rule out the possibility that the observed decreases in spatial specificity of functional communities support the dedifferentiation hypothesis. First, the working memory performance was poorer in the older adults before we corrected for multiple covariates. This is consistent with the prior studies showing working memory deficits in older adults [55]. Given the small sample size, the loss of statistical power with more covariates could have resulted in a false negative outcome. Second, these results may be pointing to early signs of deleterious brain aging that will result in future, problematic cognitive decline. Beyond this, it is likely that healthy aging reflects a combination of the two hypotheses, with the extent and patterns of altered connectivity making the difference between levels of cognitive maintenance over time. Previous research has demonstrated a relationship between both increased and decreased patterns of functional connectivity and cognitive decline [9]. A longitudinal study design, tracking maintenance of working memory performance across late adulthood in various social drinking lifestyles, would provide further insight to whether these changes are positive or negative. However, this study helps provide a benchmark for brain functioning in a healthy aging population, as well as the associated impact of a lifestyle including moderate-heavy alcohol consumption on older age.

When interpreting these data it is important to consider environmental influences that are difficult to control in an experimental setting [56]. For example, the lifestyle-cognition hypothesis holds that maintaining an active lifestyle and engaging in certain activities may help to prevent cognitive decline. Research findings have shown that older adults with high cognitive function participate in certain lifestyle characteristics such as socializing, volunteering, and leisure activity with greater frequency [57, 58]. The older participants in this study were relatively healthy, community-dwelling adults in addition to light or moderate-heavy alcohol consumers. It is possible that these lifestyle factors are more prevalent in this population and may be contributing to these age related changes in brain functioning.

Research limitations include a relatively small sample size when compared to previous research based on large-scale epidemiological studies that explored volumetric brain differences in older adult drinkers. To compensate for this, a great deal of attention was paid to screening and recruitment of participants. Participants were healthy community dwelling adults, who consumed alcohol within ranges most common to the older American adult demographic. Moreover, a great deal of attention was paid to the quantity and frequency of alcohol consumption so as to represent a truly moderate-heavy consumption pattern, and all adults were screened for signs of alcohol or substance use/abuse. Together, these features helped to make study findings more ecologically relevant to today’s older adult population [59]. It should be noted that the older adult group included participants with medical conditions (i.e. hypertension, diabetes, depression) and medication regimens that may have influenced the results of this study (see Table 2). Exclusionary criteria controlled for severe cases of these conditions or for those who had not achieved long term stabilization with medical treatment (for details see Screening section). Future research in this field is encouraged and would benefit from studies adopting a longitudinal design so as to better address questions of causality. Studies that address other features of network structure and that further explore the nature of observed connectivity changes in the CEN and DMN will also help elucidate the effects of social alcohol consumption on brain health in older adults.

To conclude, a lifestyle of moderate-heavy alcohol consumption in older age was associated with greater age-related alterations in CEN connectivity during a working memory task. Moderate-heavy alcohol consumption was not associated with changes in DMN connectivity, nor were any differences in whole-brain summaries of functional network topology observed. The decrease in CEN community consistency may reflect compensatory connectivity patterns that help maintain cognitive performance levels in those with increased alcohol exposure or could be an early sign of decline that precedes cognitive deficits. Further research is required to investigate these compensatory connectivity patterns as well as to determine whether or not this finding is negative and a sign of accelerated brain aging due to social alcohol consumption in older age.
